# Radiographic Assessment of Spinopelvic Parameters in Asymptomatic Ethiopian Adults: A Cross-Sectional Study

**DOI:** 10.2106/JBJS.OA.26.00028

**Published:** 2026-06-09

**Authors:** Fentahun Bantigegn Seyoum, Alazar Menbere Haile, Eskinder Kebede Tadesse, Kaleab Tesfaye Reda, Tofike Aman Kersema, Semehal Berihun Demewez, Amanuel Berhaneselassie Stephano, Michael Mamusha Shenkute, Biruk Lambisso Wamisho

**Affiliations:** 1 Department of Orthopedics and Trauma Surgery, Tikur Anbessa Specialized Hospital, College of Health Sciences, Addis Ababa University, Addis Ababa, Ethiopia; 2 Department of Orthopedics and Trauma Surgery, School of Medicine, College of Medicine and Health Sciences, University of Gondar, Gondar, Ethiopia

## Abstract

**Background:**

Spinopelvic alignment is central to spinal biomechanics and surgical planning. Normative spinopelvic parameters vary by geography and ethnicity, and reference data for Ethiopia have not previously been reported.

**Objectives:**

This study aimed to determine normative spinopelvic parameters in healthy Ethiopian adults and examine their variation based on age and sex.

**Methods:**

An observational cross-sectional study was conducted at Tikur Anbessa Specialized Hospital between August and December 2025. The study included 117 healthy adults (60 men, 57 women). Standardized standing lateral lumbosacral radiographs were obtained. Two spine surgeons independently measured pelvic incidence (PI), sacral slope (SS), pelvic tilt (PT), and lumbar lordosis (LL).

**Results:**

The median age was 40 years. The mean values were PI 52.6° ± 8.9°, SS 39.8° ± 7.4°, PT 12.9° ± 7.0°, and LL 60.1° ± 8.5° (measured from the inflection point to S1). LL exceeded values reported in Asian and North American cohorts but was comparable with African and European populations. Women demonstrated higher values across all parameters. Multivariable regression identified PI (β = 0.83, p < 0.001), PT (β = −0.76, p < 0.001), and female sex (β = 3.05, p = 0.008) as independent predictors of LL (R^2^ = 0.64).

**Conclusions:**

This study establishes the first normative spinopelvic reference values for asymptomatic Ethiopian adults, providing a foundation for population-specific alignment assessment and future outcome studies.

**Level of Evidence:**

Level IV Diagnostic. See Instructions for Authors for a complete description of levels of evidence.

## Introduction

Spinopelvic alignment describes the coordinated orientation of the spine and pelvis that enables maintenance of an upright posture within the “cone of economy”^1^. This relationship is quantified using key radiographic parameters, such as pelvic incidence (PI), sacral slope (SS), pelvic tilt (PT), and lumbar lordosis (LL). PI is a fixed anatomical reference that reflects individual pelvic morphology, whereas PT and SS are dynamic variables that adjust with posture and serve as compensatory mechanisms in response to structural spinal changes or age-related degeneration^2,3^. Historically, the difference between PI and LL has been used as a reliable metric for planning spinal deformity correction^4^. Achieving the desired alignment has shown strong associations with postoperative clinical outcomes and health-related quality of life^5-7^.

Normative spinopelvic parameters vary by age, sex, and ethnicity. Lukas et al. reported smaller LL, SS, and PI in Asians and larger LL, PT, and PI in African Americans, highlighting population-specific differences in spinopelvic alignment^8^.

Although reference values exist for Western and Asian populations, African data remain limited^8,9^. Most studies rely on admixed African American cohorts or small regional samples, with only one study conducted on an asymptomatic population from sub-Saharan Africa^10-14^. Given documented ethnic and regional variability, applying nonrepresentative reference standards may result in misclassification of sagittal alignment and inappropriate surgical targets^15,16^.

Normative values for healthy Ethiopian adults have not been established, and surgeons often rely on nonlocal data that may not reflect population-specific anatomy or compensatory patterns. This study aims to define normative spinopelvic parameters in asymptomatic Ethiopian adults and examine variation by age and sex.

## Materials and Methods

### Study Design and Setting

After approval of the study protocol by the Department of Orthopedics and Trauma Surgery Research Ethics Review Committee (Ref. No. MF/ORTH/488/2025), a cross-sectional observational study was conducted at Tikur Anbessa Specialized Hospital (TASH) between August and December 2025. TASH is a national referral hospital that serves a wide geographic area.

### Participants

Asymptomatic Ethiopian adults aged 18 to 75 years were recruited consecutively during the study period from our outpatient clinics. These were volunteer attendants who came to our clinics accompanying other patients. While consecutive recruitment reduced investigator-driven selection bias, the convenience-based sampling may limit representativeness, as participants were drawn from individuals with access to tertiary care facilities. Written informed consent was obtained from all participants.

### Eligibility Criteria

Inclusion criteria were Ethiopian nationality, age between 18 and 75 years, self-reported absence of current or prior spine-related or hip-related symptoms, and ability to provide written informed consent and undergo standardized standing radiography. Exclusion criteria included prior spinal, pelvic, or hip surgery; clinical or radiographic evidence of spinal or hip pathology; lower-limb disorders involving the hip, knee, or ankle that could impair neutral standing posture; and radiographs of insufficient quality for accurate anatomical landmark identification.

### Sample Size and Sampling

The sample size was calculated to estimate the mean PI with 95% confidence. Based on prior normative global studies, an SD of 10° and a margin of error of 2° were assumed^8^. Using the single-mean sample size formula, a minimum of 96 participants was required. To account for potential exclusions due to incidental pathology or inadequate image quality, a 30% contingency was added, yielding a target sample size of 125 participants. All eligible and consenting adults were invited to participate.

### Demographic Data Collection

Height and weight were measured with standard clinical scales and stadiometers to calculate body mass index (BMI).

### Radiographic Protocol

All participants underwent standardized standing lateral lumbosacral radiography, with images extending superiorly to T11. Imaging was performed with participants standing upright with hips and knees fully extended. The arms were flexed anteriorly, with the hands resting on a support, to prevent obstruction of spinal landmarks.

### Radiographic Measurements

LL was measured according to the method described by Roussouly as the angle between the point of inflection from LL to thoracic kyphosis and the superior endplate of S1^17^. To ensure comparability with prior literature, LL was also measured through the traditional L1-S1 technique, defined by the superior endplates of L1 and S1. PI, SS, and PT were measured using femoral head centers as pelvic reference points.

Radiographs were analyzed using RadiAnt DICOM Viewer with calibrated angle tools. Two fellowship-trained spine surgeons independently performed all measurements on 2 separate occasions 1 week apart. Reviewers were blinded to demographics and each other’s results.

### Statistical Analysis

Statistical analysis was performed using RStudio version 2024.04.2 (R Core Team, 2024). Normality of continuous variables was checked using the Shapiro-Wilk test. Interobserver and intraobserver reliability were evaluated with intraclass correlation coefficients using a 2-way random-effects model for absolute agreement with 95% confidence intervals (CI). Descriptive statistics were reported as mean ± SD for normally distributed variables and as median with interquartile range for non-normally distributed variables. Sex-based differences were analyzed using 2-sample *t*-tests or Wilcoxon rank-sum tests as appropriate. Associations between age and spinopelvic parameters were assessed using Spearman rank correlation coefficient to accommodate the non-normal distribution of age.

Simple linear regression was used to examine relationships between PI and the other spinopelvic parameters, and multivariable linear regression was used to identify factors associated with LL. Missing data were addressed using multiple imputation by chained equations. Forty data sets were generated using predictive mean matching, and estimates were pooled using Rubin rules. A complete-case analysis (n = 86) was performed as a sensitivity analysis.

Regression model assumptions were verified by assessing multicollinearity through variance inflation factors (VIF <5), normality of residuals using the Shapiro-Wilk test, and homoscedasticity through the studentized Breusch-Pagan test. A p-value < 0.05 was considered statistically significant. The STROBE (Strengthening the Reporting of Observational Studies in Epidemiology) guidelines were used in producing this article.

## Results

**Fig. 1:**
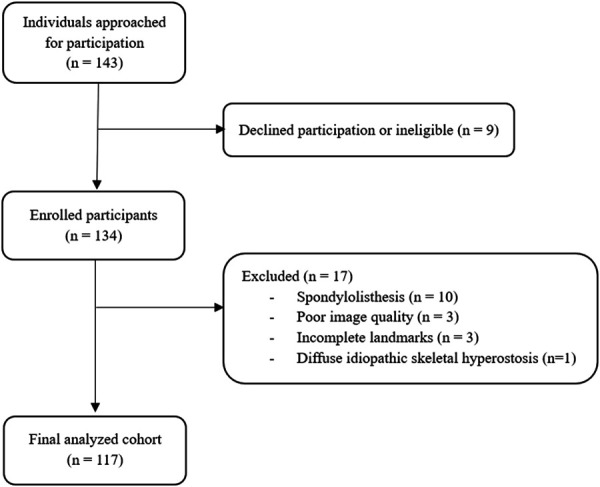
Participant enrollment and exclusion criteria.

Initially, 134 participants were enrolled in the study. Seventeen were excluded: 10 for incidental radiographic spondylolisthesis, 3 for inadequate visualization of pelvic or spinal landmarks, 3 for incomplete capture of required anatomical landmarks, and 1 for diffuse idiopathic skeletal hyperostosis. The final cohort included 117 participants (60 men and 57 women), with ages ranging from 18 to 73 years and a median age of 40 years (interquartile range [IQR], 30-51). See Figure 1 for a flow diagram of the enrollment process.

Inter- and intraobserver agreement for all spinopelvic parameters was good to excellent, with intraobserver interclass correlation coefficients (ICCs) ranging from 0.89 to 0.97 (Table S1) and interobserver ICCs ranging from 0.79 to 0.92 (Table S2). SS, PI, and LL were normally distributed, whereas PT was right-skewed.

The mean PI was 52.6° (SD 8.9) and the mean SS was 39.8° (SD 7.4), while the median PT was 12.1° (IQR, 8.1°-16.5°). LL measured from the inflection point to S1 had a mean of 60.1° (SD 8.5), compared with 59.2° (SD 9.2) using the L1-S1 method. The 2 LL measurement methods demonstrated excellent agreement (ICC = 0.94; mean difference 1.0°, p < 0.001). Age-stratified normative spinopelvic parameters are presented in Table S3.

According to the theoretical Roussouly classification, 10.3% (n = 12) of patients exhibited a type 1 curve, 7.7% (n = 9) a type 2 curve, 59.0% (n = 69) a type 3 curve, and 23.1% (n = 27) a type 4 curve. PI, SS, and LL increased progressively across theoretical Roussouly types (all p < 0.001), with LL scaling proportionally to PI (Table S4). The PI-LL mismatch approached neutrality in higher PI types (p < 0.001; Table S4).

Sex-based comparisons demonstrated significantly higher values among female participants for all measured parameters. Compared with men, women demonstrated greater SS (41.7° vs. 37.9°, p = 0.006), PT (13.9° vs. 11.1°, p = 0.011), PI (56.1° vs. 49.3°, p < 0.001), and LL (63.7° vs. 56.8°, p < 0.001) (Table I).

**TABLE I:** Comparison of Spinopelvic Parameters By Sex

Variable	Female (n = 57)	Male (n = 60)	Difference (95% CI)	Effect Size	p
Sacral slope (°)	41.7 (7.5)	37.9 (6.9)	3.7 (1.1-6.4)	Cohen’s d = 0.52	**0.006**
Pelvic tilt (°)	13.9 (10.1-17.9)	11.1 (7.2-14.3)	3.0 (0.7-5.3)	Rank-biserial = 0.23	**0.011**
Pelvic incidence (°)	56.1 (7.7)	49.3 (8.8)	6.8 (3.8-9.8)	Cohen’s d = 0.82	**<0.001**
Lumbar lordosis (°)	63.7 (7.2)	56.8 (8.3)	6.9 (4.1-9.8)	Cohen’s d = 0.89	**<0.001**

IQR = interquartile range.

Values are mean (SD) for PI, LL, and SS; pelvic tilt is median (IQR). LL was measured from the inflection point to S1.

Group comparisons used pooled-variance Student *t*-test (PI, LL, SS) and Wilcoxon rank-sum test (PT).

Effect sizes are Cohen d for normally distributed variables and rank-biserial correlation for pelvic tilt.

Bold values indicate statistically significant results (p < 0.05)

Age demonstrated a modest positive correlation with PT (rho = 0.31, p < 0.001), showing a small to moderate effect size and explaining ∼10% of the variability in PT. No statistically significant correlations were observed between age and LL (rho = 0.13, p = 0.17), age and SS (rho = 0.04, p = 0.70), or age and PI-LL mismatch (rho = 0.16, p = 0.09). Interparameter correlation revealed a strong association between SS and LL, while PI showed moderate correlations with SS, PT, and LL. The association between SS and PT was weak (Table II).

**TABLE II:** Correlation Between Spinopelvic Parameters

Relationships	Spearman ρ	Strength of Correlation	p*
PI ↔ SS	0.62	Moderate	**<0.001**
PI ↔ PT	0.55	Moderate	**<0.001**
PI ↔ LL	0.56	Moderate	**<0.001**
SS ↔ LL	0.78	Strong	**<0.001**
SS ↔ PT	-0.24	Weak	**0.010**
PT ↔ LL	-0.06	Very weak	0.511

* Spearman correlation.

Bold values indicate statistically significant results (p < 0.05)

Bivariate linear regression using PI as the independent variable confirmed that PI significantly predicted the other spinopelvic parameters. PI accounted for 41.8% of the variance in SS, 34.3% in PT, and 34.7% in LL. The regression equations are:

$$\text{SS}=0.538\times \text{PI}+11.446\;\left(R^{2}=0.418,p<0.001\right)$$

$$\text{PT}=0.459\times \text{PI}\;‐\;11.238\;\left(R^{2}=0.343,p<0.001\right)$$

$$\text{LL}=0.562\times \text{PI}+30.538\;\left(R^{2}=0.347,p<0.001\right)$$

Six observations exceeded the Cook’s distance threshold (4/n), but none showed undue influence (maximum Cook’s D = 0.233).

In the primary multivariable model using multiple imputation, PI (β = 0.83, p < 0.001), PT (β = −0.76, p < 0.001), and female sex (β = 3.05, p = 0.008) were independently associated with LL (pooled R^2^ = 0.64). BMI and age were not significant predictors (Table III). Sensitivity analysis using complete case data (n = 86) yielded broadly similar results, with PI and PT remaining significant predictors of LL. BMI reached statistical significance in the complete case model (β = 0.35, p = 0.008), whereas the association with female sex was attenuated and no longer statistically significant. Given the smaller sample size in the complete case analysis, the model may have been underpowered to detect modest associations, and these findings should be interpreted cautiously.

**TABLE III:** Multivariable Linear Regression Predicting Lumbar Lordosis

	Multiple Imputation (Primary)			Complete Case (Sensitivity)		
Predictor	Estimate (SE)	95% CI	p	Estimate (SE)	95% CI	p
Intercept	19.92 (4.56)	10.99 to 28.86	**<0.001**	16.45 (4.84)	6.97 to 25.93	**0.001**
Body mass index	0.17 (0.15)	−0.11 to 0.46	0.245	0.35 (0.13)	0.10 to 0.61	**0.008**
Pelvic incidence	0.83 (0.07)	0.69 to 0.97	**<0.001**	0.85 (0.08)	0.69 to 1.01	**<0.001**
Pelvic tilt	−0.76 (0.09)	−0.94 to −0.59	**<0.001**	−0.85 (0.10)	−1.05 to −0.66	**<0.001**
Age (yr)	0.02 (0.04)	−0.06 to 0.10	0.586	0.02 (0.05)	−0.08 to 0.11	0.752
Sex (female)	3.05 (1.12)	0.85 to 5.25	**0.008**	1.70 (1.31)	−0.87 to 4.27	0.199
Model diagnostics						
Observations	117			86		
Pooled R^2^	0.64	0.52 to 0.74				
R^2^				0.67		
Adjusted R^2^				0.65		
RMSE				4.89		

CI = confidence interval.

Bold values indicate statistically significant results (p < 0.05).

Coefficients and pooled R from the multiple imputation model were combined using Rubin rules. Complete case estimates are presented as a sensitivity analysis.

Compared with published normative data sets from Africa, Asia, Europe, North, and South America (Table IV), the cohort mirrored European samples in PI (52.6° vs. 52.6°) and SS (39.8° vs. 39.5°). LL (59.2°-60.1°) was comparable with African and European averages but substantially exceeded those of Asia (45.8°) and North America (44.4°)^8,12^.

**TABLE IV:** Regional Summary of Spinopelvic Parameters Synthesized From Literature

Parameter	Region	No. of Cohorts	Total N	Mean ± SD (°)	95% CI
Sacral slope (°)					
	African (current study)		117	39.8 ± 7.4	(38.4-41.1)
	African (Congolese)	1	212	41.0 ± 8.4	(39.9-42.1)
	African American	1	36	41.4 ± 9.2	(38.4-44.4)
	Asian	58	5,588	34.2 ± 8.8	(34.0-34.4)
	European	13	1,268	39.5 ± 7.4	(39.1-40.0)
	Middle Eastern	5	374	38.1 ± 8.1	(37.3-38.9)
	North American	9	880	39.3 ± 8.6	(38.7-39.9)
	South American	2	137	36.6 ± 6.6	(35.5-37.7)
Pelvic tilt (°)					
	African (current study)		117	12.9 ± 7.0	(11.7-14.2)
	African (Congolese)	1	212	13.2 ± 6.5	(12.3-14.1)
	African American	1	36	15.9 ± 7.0	(13.6-18.2)
	Asian	62	7,340	14.5 ± 8.6	(14.3-14.7)
	European	13	1,268	12.5 ± 6.6	(12.2-12.9)
	Middle Eastern	5	374	10.3 ± 7.1	(9.6-11.0)
	North American	8	874	13.5 ± 7.3	(13.0-14.0)
	South American	2	137	10.1 ± 7.0	(9.0-11.3)
Pelvic incidence (°)					
	African (current study)		117	52.6 ± 8.9	(51.0-54.2)
	African (Congolese)	1	212	54.4 ± 7.8	(53.4-55.4)
	African American	1	36	57.7 ± 11.5	(53.9-61.5)
	Asian	58	6,798	48.0 ± 11.2	(47.8-48.3)
	European	11	955	52.6 ± 9.7	(52.0-53.2)
	Middle Eastern	5	374	48.5 ± 8.1	(47.7-49.4)
	North American	9	880	51.0 ± 12.5	(50.2-51.8)
	South American	2	137	46.8 ± 9.2	(45.2-48.3)
Lumbar lordosis (°)					
	African (current study)		117	59.2 ± 9.2	(57.5-60.8)
	African (Congolese)	1	212	61.1 ± 9.7	(59.8-62.4)
	African American	1	36	57.2 ± 13.2	(52.9-61.5)
	Asian	62	7,258	45.8 ± 13.1	(45.5-46.1)
	European	11	1,006	58.3 ± 11.5	(57.6-59.0)
	Middle Eastern	3	236	53.7 ± 13.6	(51.9-55.4)
	North American	9	880	44.4 ± 12.9	(43.6-45.3)
	South American	2	137	51.4 ± 10.8	(49.6-53.2)
PI-LL mismatch					
	African (current study)		117	−6.5 ± 8.6	(−8.1 to −5.0)
	African (Congolese)	1	212	−6.7 ± 8.0	(−7.8 to −5.6)
	African American	1	36	0.5 ± 11.2	(−3.1 to 4.1)
	Asian	55	6,557	2.1 ± 12.5	(1.8 to 2.4)
	European	10	853	−5.1 ± 10.4	(−5.8 to −4.4)
	Middle Eastern	3	236	−6.8 ± 11.2	(−8.3 to −5.4)
	North American	9	880	6.6 ± 12.0	(5.8 to 7.4)
	South American	2	137	−4.6 ± 9.0	(−6.1 to −3.1)

Values are sample-size weighted means; SDs represent pooled within-study and between-study variance.

Reference data synthesized from individual studies reported in Lukas, Kenneth et al., 2023; Arima, Hideyuki et al., 2018, and Mukaya et al., 2016.

## Discussion

This study provides the first population-specific normative spinopelvic reference values for asymptomatic Ethiopian adults. The mean PI of 52.6° ± 8.9°, SS of 39.8° ± 7.4°, and PT of 12.9° ± 7.0° conform to the geometric identity PI = PT + SS and place this cohort within an intermediate PI range compared with previously reported populations^8,9^. PI significantly predicted SS, PT, and LL, explaining 34% to 42% of their variance. This is consistent with previous studies and reflects the multifactorial nature of sagittal alignment^10^.

Sex differences were observed across all spinopelvic measures, with women demonstrating higher PI, SS, PT, and LL. These findings are consistent with the recent reports by Nakano et al.^18^. The effects of age were relatively modest. While PT showed a mild increase with age, SS and LL measurements did not exhibit significant age-related trends.

The strong correlation between SS and LL in our cohort reinforces their biomechanical coupling and supports Dubousset's chain-of-balance concept^19,20^. The inverse relationship between SS and PT further supports their complementary roles in maintaining global sagittal alignment^21^.

In multivariable regression analysis, PI, PT, and sex remained independently associated with LL, whereas age was no longer a significant predictor after adjustment. Inclusion of PT significantly improved model performance, increasing the adjusted R^2^ from 0.40 to 0.64, with a significant nested model comparison (F = 69.6, p < 0.001). This finding supports PT as a posture-dependent compensatory alignment parameter reflecting pelvic orientation beyond fixed pelvic morphology, rather than a redundant correlate of PI.

Restoring harmonious spinopelvic alignment is central to deformity correction^22^. Strategies include aligning a patient’s current Roussouly with its theoretical type and addressing SRS-Schwab classification modifiers.

The Roussouly classification describes 4 sagittal morphologic patterns associated with distinct alignment strategies^23^. Types 1 and 2 generally require less restoration of LL than types 3 and 4^23^. Deviation from the theoretical Roussouly type in adult spinal deformity has been associated with higher complication rates and poorer quality of life^5,24^. In our cohort, type 3 was the most common morphology, similar to the MEANS study (59.0% vs. 55.9%)^25^.

PI-LL mismatch, introduced by Schwab et al., is another widely used measure to assess lumbopelvic harmony, with a threshold of approximately 10° associated with improved outcomes^26^. Recent studies, however, suggest that rigid thresholds may oversimplify alignment goals, highlighting the need for population-specific values of PI^27,28^.

By providing the first anatomical reference data for the Ethiopian population, these normative data enable surgeons to tailor alignment targets according to the population-specific distribution of PI and Roussouly types. However, because PI-LL mismatch alone does not fully reflect global sagittal alignment, the absence of parameters such as sagittal vertical axis and T1 pelvic angle limits the direct clinical applicability of these findings.

### Limitations of the Study

First, the use of a single-center convenience sample of clinic attendants introduces potential selection and regional bias and may not represent the healthy general population. Furthermore, the lack of an internal control group necessitated comparisons to be made with historical data, which could further limit the generalizability of the findings. Second, the asymptomatic status of participants was determined through self-reporting and clinical interviews rather than using validated questionnaires. Furthermore, important demographic covariates were not systematically collected for all participants, which limits the interpretability of the results. Third, the clinical relevance of these findings is limited by the absence of global sagittal alignment parameters, which could not be measured due to a lack of full-spine imaging capabilities at our facility. In addition, the modest sample size of our cohort limits the robustness of the proposed reference values. Finally, this study relied on static standing radiographs; however, spinal alignment is a dynamic process, and future research should incorporate dynamic assessments to better characterize alignment in functional positions^29,30^.

## Conclusion

These Ethiopian population-specific reference values provide an anatomical benchmark for contextualizing alignment targets and informing future research.

## Funding

No funding was received.

## Appendix

Supporting material provided by the authors is posted with the online version of this article as a data supplement at jbjs.org (http://links.lww.com/JBJSOA/B195). This content was not copyedited or verified by JBJS.
